# Evaluation of neurotrophic factors and education level as predictors of cognitive decline in alcohol use disorder

**DOI:** 10.1038/s41598-021-95131-2

**Published:** 2021-08-02

**Authors:** Nerea Requena-Ocaña, Pedro Araos, María Flores, Nuria García-Marchena, Daniel Silva-Peña, Jesús Aranda, Patricia Rivera, Juan Jesús Ruiz, Antonia Serrano, Francisco Javier Pavón, Juan Suárez, Fernando Rodríguez de Fonseca

**Affiliations:** 1 Mental Health Clinical Management Unit, Institute of Biomedical Research of Malaga-IBIMA, Regional University Hospital of Málaga, 29010 Málaga, Spain; 2 School of Psychology, Complutense University of Madrid, Madrid, Spain; 3 Department of Psychobiology and Methodology of Behavioral Sciences, School of Psychology, University of Málaga, 29010 Málaga, Spain; 4 School of Medicine, University of Málaga, 29071 Málaga, Spain; 5 Provincial Drug Addiction Center of Málaga, Provincial Council of Málaga, Málaga, Spain; 6 Cardiac Clinical Management Unit, IBIMA, University Hospital Virgen de la Victoria, 29010 Málaga, Spain; 7 Department of Human Anatomy, Legal Medicine and History of Science, IBIMA, Facultad de Medicina, University of Málaga, Bulevar Louis Pausteur, 29071 Málaga, Spain; 8 Laboratorio de Investigación, IBIMA, Hospital Universitario Regional de Málaga, Avenida Carlos Haya 82, 29010 Málaga, Spain

**Keywords:** Neuroscience, Psychology, Biomarkers

## Abstract

Cognitive reserve (CR) is the capability of an individual to cope with a brain pathology through compensatory mechanisms developed through cognitive stimulation by mental and physical activity. Recently, it has been suggested that CR has a protective role against the initiation of substance use, substance consumption patterns and cognitive decline and can improve responses to treatment. However, CR has never been linked to cognitive function and neurotrophic factors in the context of alcohol consumption. The present cross-sectional study aims to evaluate the association between CR (evaluated by educational level), cognitive impairment (assessed using a frontal and memory loss assessment battery) and circulating levels of brain-derived neurotrophic factor (BDNF) and neurotrophin-3 (NT-3) in patients with alcohol use disorder (AUD). Our results indicated that lower educational levels were accompanied by earlier onset of alcohol consumption and earlier development of alcohol dependence, as well as impaired frontal cognitive function. They also suggest that CR, NT-3 and BDNF may act as compensatory mechanisms for cognitive decline in the early stages of AUD, but not in later phases. These parameters allow the identification of patients with AUD who are at risk of cognitive deterioration and the implementation of personalized interventions to preserve cognitive function.

## Introduction

Cognitive decline is currently considered a global health problem. The World Health Organization (WHO) recognizes dementia as a public health priority, positioning it as one of the main diseases of the twenty-first century. The prevalence of dementia is predicted to markedly increase in the next 30 years, affecting 131.5 million people by 2050^1^. Therefore, the WHO has endorsed a *global action plan on the public health response to dementia 2017–2025* for the development of a coordinated global response to effectively address the issue of this disease^2^.

Although high alcohol consumption has been associated with an acceleration of cognitive decline in aging^3^, the WHO considers alcohol consumption to be one of the many risk factors for dementia and not a main component in the etiology of the disease. However, Schwarzinger et al.^4^ found that alcohol use disorder (AUD) is the most robust risk factor for the onset of any type of dementia, especially early onset dementia. More strikingly, in a recent epidemiological study, the most common diagnosis in cases of early-onset dementia was alcohol-related dementia (18.4%), followed by Alzheimer's disease (17.7%), vascular dementia (12.8%) and frontotemporal dementia (11.3%)^5^. In addition, differences in alcohol consumption habits have been shown to affect the risk of dementia in the absence of genetic factors explaining these associations^6^.

Regarding preventive mechanisms, the concept of cognitive reserve has been developed to explain the discrepancy between the extent of brain pathology and the expected cognitive performance of an individual^7^. Cognitive reserve is understood as an active mechanism to address brain damage that depends on the creation of flexible and effective neural networks as a result of cognitive stimulation throughout life^8^. Thus, cognitive reserve seems to be the result of innate intelligence, years of schooling, occupation, life experiences, social relationships and leisure activities^9–11^. However, one of the most commonly used measures of cognitive reserve is years of education because it allows the other factors to be inferred^12^. For example, it has been shown that a high education level softens the clinical manifestations and the progression of mild cognitive impairment^13^; in addition, it has been related to fewer alterations in the biomarkers t-tau, p-tau, t-tau/AB42, and p-tau/AB42 in individuals with Alzheimer's disease^14^.

Thus, there is evidence that a greater reserve can act as a protective factor against normal aging processes^15^; the appearance of neuropathological diseases, such as mild cognitive impairment^16^, Alzheimer’s disease^17,18^, Parkinson's disease^19^, multiple sclerosis^20^, brain damage related to trauma, stroke or tumor^9,21^; and psychiatric disorders, such as depression, bipolar disorder, anxiety and psychosis^22,23^. In addition, the concept of cognitive reserve has recently been established in the field of addictions, and research suggests that it has a preventive role against the onset of drug use and is associated with less severe consumption patterns, less cognitive decline and better response to treatment^24^.

Along these lines, it is widely known that chronic alcohol consumption can affect the central nervous system (CNS) and cause the deterioration of cognitive functions^25^. This deterioration has a neurodegenerative component that is due not only to the nutritional deficiencies (i.e., thiamine deficiency) of chronic drinkers but also to neurotoxicity mediated by inflammatory phenomena that are directly activated by alcohol, including the activation of TL4 receptors, the release of proinflammatory cytokines and the onset of oxidative stress^26,27^. These phenomena involve the activation of the brain’s resident immune system through microglial cells and astrocytes, which leads to the emergence of neuronal apoptosis and even necrosis in the central nervous system^28,29^. Postmortem studies have revealed a significant loss of gray and white matter in the brains of people with AUD, especially in the prefrontal cortex and cerebellum^30^.

Regarding cognitive integrity, patients who have chronic alcohol consumption show abnormalities in motor control, processing speed, sustained attention, memory and learning and overall executive function^3,31,32^, with visuospatial cognition being the most affected component^33,34^. Specifically, patients with AUD show deficits in tasks that require cognitive control, cognitive flexibility, inhibition, planning and working memory^35,36^. Additionally, these patients exhibit changes in emotional processing and social cognition^37^. It is important to mention that AUD is often accompanied by other substance use disorders and psychiatric disorders that, when added to cognitive deficits, severely worsen the patient’s clinical situation, leading to even greater social stigmatization, withdrawal from health services and, ultimately, social exclusion^25,38,39^.

On the other hand, growth factors are a set of proteins that play a crucial role in cell growth, proliferation and differentiation in the CNS and are important to cognitive processes^40^. Among the neurotrophic factors are neurotrophins, including brain-derived neurotrophic factor (BDNF), neurotrophin 3 (NT-3) and neurotrophin 4. The functions attributed to BDNF include the regulation of neurogenesis, synaptogenesis and gliogenesis and the control of long-term potentiation (LTP) mechanisms in the hippocampus that result in increases in memory and cognition^41^. Clinical studies have described the decrease in BDNF levels and cognitive decline in patients with psychiatric disorders such as depression, schizophrenia and bipolar disorder^42^ and in patients with neuropathological diseases such as epilepsy^43^, mild cognitive impairment, Alzheimer’s disease^44^ and Parkinson's disease^45^. In addition, our group has recently associated BDNF with the cognitive deterioration of patients with AUD during abstinence^46^. NT-3 has been associated with proliferation, migration and neuronal differentiation, although it does not appear to be involved in CNS maturation, and little is known about its role in cognition^47^. In a preclinical model, deregulated NT-3 expression was found in both the neonatal brain and the adult hippocampus, which could explain the cognitive deficits of Down syndrome^48^. In a clinical study, the rs6332 polymorphism of NT-3 was associated with executive function in patients with Alzheimer’s disease^49^. Additionally, insulin-like growth factors (IGF1 and IGF2) are another family of neurotrophic proteins involved in the regulation of the proliferation, development and growth of neuronal cells, whose circulation is decreased in patients with AUD^50^.

Given that the longevity of the human population is increasing and that the data on alcohol consumption continue to be alarmingly high, in the present study, we wanted to identify the impact of cognitive reserve (evaluated by educational level) and the circulating neurotrophic factors as decisive elements in the onset and progression of cognitive decline in patients diagnosed with AUD who attend outpatient treatment. To this end, we conducted a cross-sectional study to evaluate the associations between education level (primary, secondary and university), the presence of cognitive decline and circulating levels of neurotrophic factors BDNF, NT-3, IGF-1, IGF-2 and its associated protein IGFBP-3 in alcohol-abstinent patients with AUD.

## Results

### Sociodemographic characteristics of the sample

Table 1 presents the sociodemographic description of the total sample of alcohol-abstinent patients and the subcohort with a complete neuropsychological battery (FAB and MMSE), all of whom were selected from outpatient programs for AUD. Of the total study sample, 74.4% were men, and 25.4% were women; their mean age was 49.23 years (SD = 8.87), and their body mass index was 25.99 kg/m^2^ (SD = 4.36). A total of 28.2% of the population was single, 37.8% was married or had a partner, and 30.9% was divorced or separated. Regarding educational level, 37% had a primary education, 42.2% had a secondary education, and 16.8% had a university education. A total of 24% were employed, compared to 45.4% who were unemployed, and 30.5% were on sick leave, were retired or were homemakers. As Table 1 shows, the subcohort of patients had sociodemographic characteristics that were very similar to those of the total sample, indicating that it was a representative subcohort for this group of variables.

**Table 1:** Sociodemographic characteristics of the total sample of patients.

Variables	Patients with alcohol use disorder				
	Total sample N = 262	Subcohort^a^ N = 58	Statistics	df	p-value
Age (mean ± SD) (years)	49.23 ± 8.87	49.57 ± 8.20	− 0.270^b^	318	0.787
Body mass index [mean (SD)] (kg/m^2^)	25.99 ± 4.36	25.86 ± 3.81	0.210^b^	318	0.819
**Sex [N (%)]**					
Women	67(25.4)	11 (19)	1.125^c^	1	0.316
Men	195 (74.4)	47 (81)			
**Marital status [N (%)]**					
Single	74 (28.2)	14 (24.1)	2.696^c^	4	0.610
Cohabiting	99 (37.8)	20 (34.5)			
Separated	81 (30.9)	22 (37.9)			
Widow	8 (3.1)	2 (3.4)			
**Education level [N (%)]**					
Elementary	97 (37)	22 (37.9)	0.240^c^	2	0.887
Secondary	121 (46.2)	25 (43.1)			
University	44 (16.8)	11 (19)			
**Occupation [N (%)]**					
Employed	63 (24)	11 (19)	2.416^c^	4	0.660
Unemployed	119 (45.4)	30 (51.7)			
Other	80 (30.5)	17 (29.3)			

*df* degree of freedom.

^a^Patients with neuropsychological battery (FAB and MMSE).

^b^Chi-square test statistic.

^c^Student’s t test statistic.

### Variables associated with alcohol consumption

The variables related to alcohol consumption in the total AUD group and the subcohort with a complete neuropsychological battery (FAB and MMSE) were evaluated and are described in Table 2. Of the total study sample, the mean age at the start of alcohol consumption was 16.26 years (SD = 3.9), and the mean age when dependence developed was 29.56 years (SD = 10.62). The mean number of alcohol addiction severity criteria (range 0–11) was 7.46 (SD = 2.19), the mean duration of abstinence from alcohol at the time of evaluation was 306.67, and the mean length of AUD diagnosis was 15.35 years (SD = 10.41). The AUD group had a high prevalence of other substance use disorders (40.5%), with cocaine use disorder being the most prevalent (29.4%). In addition, a high prevalence of other psychiatric disorders was observed (68.3%). Thus, in the AUD group, 45.4% and 39.3% were diagnosed with mood and anxiety disorders, respectively, at some point in their lives. The alcohol-abstinent patients had received the following psychotropic medications for at least 12 months: antidepressants (43.5%), anxiolytics (39.3%) and anticraving medications (27.1%). Lastly, 64.1% of the patients were treated with disulfiram. As Table 1 shows, the subcohort of patients with the complete neuropsychological battery (FAB and MMSE) has some sociodemographic characteristics that were very similar to those of the total sample, indicating that they were representative in terms of the variables related to alcohol use and psychiatric comorbidity. The patients in the subcohort differed from the total sample only in that they had a shorter duration of abstinence at the time of evaluation (128.61 days; t_312_ = 2.443, *p* = 0.003).

**Table 2:** Variables associated with alcohol consumption and psychiatric comorbidity.

Variables	Patients with alcohol use disorder				
	Total sample N = 262	Subcohort N = 58	Statistics^a^	df	*p*-value
Age at onset of consumption [mean (SD)] (years)	16.26 (3.9)	15.17 (2.95)	1.670^b^	314	0.096
Age at the development of dependence [mean (SD)] (years)	29.56 (10.62)	31.23 (11.07)	− 0.946^b^	347	0.339
Length of AUD diagnosis [mean (SD)] (years)	15.35 (10.41)	16.36 (14.93)	− 0.051^b^	295	0.964
**Severity criteria [mean (range)]**					
Criteria (0–11)	7.46 (2.19)	7.88 (2.08)	− 1.285^b^	316	0.200
Duration of abstinence [Mean (mode)] (Days)	306.67 (60)	128.61 (60)	2.443^b^	312	**0.003**
**Comorbid substance use disorders [N (%)]**					
Cocaine	77 (29.4)	19 (32.8)	0.326^c^	1	0.633
Cannabis	34 (13)	6 (10.3)	0.266		0.825
Sedatives	18 (6.9)	5 (8.6)	0.245		0.579
**Comorbid psychiatric disorders N (%)]**					
Mood	119 (45.4)	25 (43.1)	0.024^c^	1	1
Anxiety	81 (30.9)	20 (34.5)	0.441		0.529
Personality	43 (16.4)	5 (8.6)	2.071		0.221
ADHD	54 (20.6)	6 (10.3)	3.028		0.217
**Psychiatric medication use [N (%)]**					
Antidepressants	114 (43.5)	31(53.4)	1.821^c^	1	0.189
Anxiolytics	103 (39.3)	19 (32.8)	0.933		0.370
Anticraving	71 (27.1)	19 (32.8)	0.713		0.420
Disulfiram use [N (%)]	168 (64.1)	45 (77.6)	0.704^c^	1	0.590

*df* degree of freedom.

Bold values are statistically significant for p < 0.05.

^a^The statistical analysis was conducted using the logarithmic transformed values to ensure that statistical assumptions were met for age at the onset of consumption, age at the development of dependency, duration AUD, severity criteria met and duration of abstinence.

^b^Student’s t test statistic.

^c^Chi-square test statistic.

### Cognitive impairment and education level in alcohol-abstinent patients

The neuropsychological evaluations of the subcohort of 58 alcohol-abstinent AUD patients showed that 75.9% had some type of cognitive impairment. Thus, 74.1% of the patients had some deficit related to frontal lobe function (evaluated with the FAB), and 36.2% of them had memory deficits (evaluated with the MFE). Specifically, 48.3% seemed to have signs of cognitive impairment similar to frontosubcortical deficit, and 25.9% had signs of impairment similar to frontosubcortical dementia, as indicated in the manual of the instrument^51^. However, 58.6% had insignificant memory problems, 19% had mild memory impairment that had little impact on daily life, and 17.2% had severe memory impairment with a significant impact on daily life, as indicated in the manual^52^.

**Figure 1:**
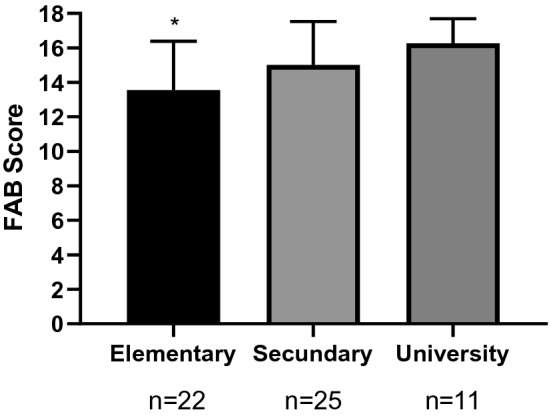
FAB scores according to education level (primary, secondary and university). There were statistically significant differences in FAB scoresbetweenthe primary and university levels of education assessed using a two-way ANCOVA with with "educational level" as a factor and “age” as covariate. The bars are estimated marginal means and 95% confidence intervals. *p < 0.05. *FAB* frontal assessment battery.

The influence of educational level on cognitive status was examined using two-way ANCOVA with “education level” as a factor and “age” as a covariate. We verified that the data met the statistical assumptions, and logarithmic transformations were performed on the variables that did not meet the assumptions. As shown in Fig. 1, there were statistically significant differences in FAB scores as a function of education level (primary, secondary, university) (F_2,58_ = 4.850, *p* = 0.012); patients with a primary education level had lower FAB scores than those with a university education level (*p* = 0.011). However, we did not find statistically significant differences in the MMFE score as a function of education level (F_2,58_ = 0.608, *p* = 0.548).

### Cognitive impairment and psychiatric comorbidity in alcohol-abstinent AUD patients

To determine whether the cognitive impairment of alcohol-abstinent AUD patients was influenced by the presence of psychiatric comorbidity, we examined the effect of mood and anxiety comorbid disorders in FAB and MMSE scores using a Student’s t test, since these were the most common psychiatric disorders in the study population (see Table 2). We did not find statistically significant differences in FAB or MMSE scores between patients with and without mood and anxiety disorders (see Supplementary Tables S1 and S2).

### Education level and variables related to alcohol consumption

The influence of education level on the variables associated with alcohol consumption was studied using ANOVA with “education level” as a factor (Table 3). We verified that the data met the statistical assumptions, and logarithmic transformations of the variables that were not normally distributed were performed. There were significant differences in the age at onset of consumption (F_2,55_ = 3.175, *p* = 0.050) and the age at the development of dependence (F_2,55_ = 4.994, *p* = 0.010) according to education level. Thus, patients with a primary education level started consuming alcohol 3.21 years earlier (*p* = 0.046) and developed alcohol dependence 11.75 years earlier than those with a university education level (*p* = 0.009).

**Table 3:** Variables related to alcohol consumption according to education level.

Variables	Subcohort (N = 58)					
	Elementary N = 22	Secondary N = 25	University N = 11	ANCOVA (statistics)^a^		
				F-value	df	*p*-value
Age at onset of consumption [mean (SD)] (years)	14.15 (2.28)	15.24 (2.09)	17.36 (4.48)	3.175	2.56	**0.050**
Age at the development of dependency [mean (SD)] (years)	26.25 (8.51)	32.24 (12.03)	38 (9.32)	4.994	2.52	**0.010**
Length of AUD diagnosis [mean (SD)] (years)	16.15 (10.93)	19.56 (18.86)	9.45 (8.25)	0.176	2.53	0.893
**Severity criteria [mean (range)]**						
Criteria	8 (2.07)	8 (2.06)	7.36 [2–11]	0.364	2.57	0.697
Duration of abstinence [mean (range)] (days)	140.95 [0–1270]	149.92 [14–149.92]	56.64 [14–120]	1.424	2.56	0.250

*df* degree of freedom.

Bold values are statistically significant for p < 0.05.

^a^Statistical analysis was conducted on the logarithmic transformed values to ensure that statistical assumptions were met.

### Cognitive impairment and variables related to alcohol consumption

To explore the effect of cognitive impairment on the variables associated with alcohol consumption, Student’s t test was used (Table 4). We verified that the data met the statistical assumptions, and logarithmic transformations of the variables that did not meet the assumptions were performed. We found statistically significant differences in the age at onset of consumption (t_54_ = 2.533, *p* = 0.014), the length of AUD diagnosis (t_51_ = -3.028, *p* = 0.004), the severity criteria of addiction (t_55_ = 2.900, *p* = 0.005) and the duration of abstinence at the time of the psychiatric evaluation (t_54_ = -3.184, *p* = 0.002). Thus, patients with cognitive impairment started consuming alcohol an average of 2 years before patients without cognitive impairment, and patients with cognitive impairment had been living with the disorder 7 years longer than patients without cognitive impairment. However, patients without cognitive impairment showed greater severity of addiction [9 vs. 7 criteria (0–11)] and a shorter duration of abstinence (158 vs. 46 days) at the time of the psychiatric evaluation.

**Table 4:** Education level and cognitive decline in variables associated with alcohol.

Variables	Subcohort (N = 57)				
	Cognitive impairment N = 43	No cognitive impairment N = 14	ANCOVA (statistics)^a^		
			F-value	df	*p*-value
Age at onset of consumption [mean (SD)] (years)	14.66 (2.64)	16.93 (3.17)	2.533	54	**0.014**
Age at the development of dependence [mean (SD)] (years)	31.37 (11.36)	30.87 (10.61)	− 0.148	56	0.883
Length of AUD diagnosis [mean (SD)] (years)	18.24 (16.30)	11.20 (8.83)	− 3.028	51	**0.004**
**Severity criteria [mean (range)]**					
Criteria	7.43 (2.03)	9.13 (1.73)	2.900	55	**0.005**
Duration of abstinence [mean (range)] (days)	157.98 [0–1440]	46.40 [14–120]	− 3.184	54	**0.002**

*df* degree of freedom.

Bold values are statistically significant for p < 0.05.

^a^Statistical analysis was conducted on the logarithmic transformed values to ensure statistical assumptions for age at onset of consumption, length of AUD diagnosis and length of abstinence.

### Education level and plasma concentrations of BDNF, NT-3, IGF-1, IGF-2 and IGFBP-3 in alcohol-abstinent AUD patients

The influence of education level on plasma levels of BDNF, 3-NT, IGF-1, IGF-2 and IGFBP-3 was evaluated using a two-way ANCOVA with "educational level" as a factor and "age" and "BMI” as covariates (see Supplementary Table S3). We confirmed that the data met the statistical assumptions, and logarithmic transformations were performed for values that did not meet the assumptions.

**Figure 2:**
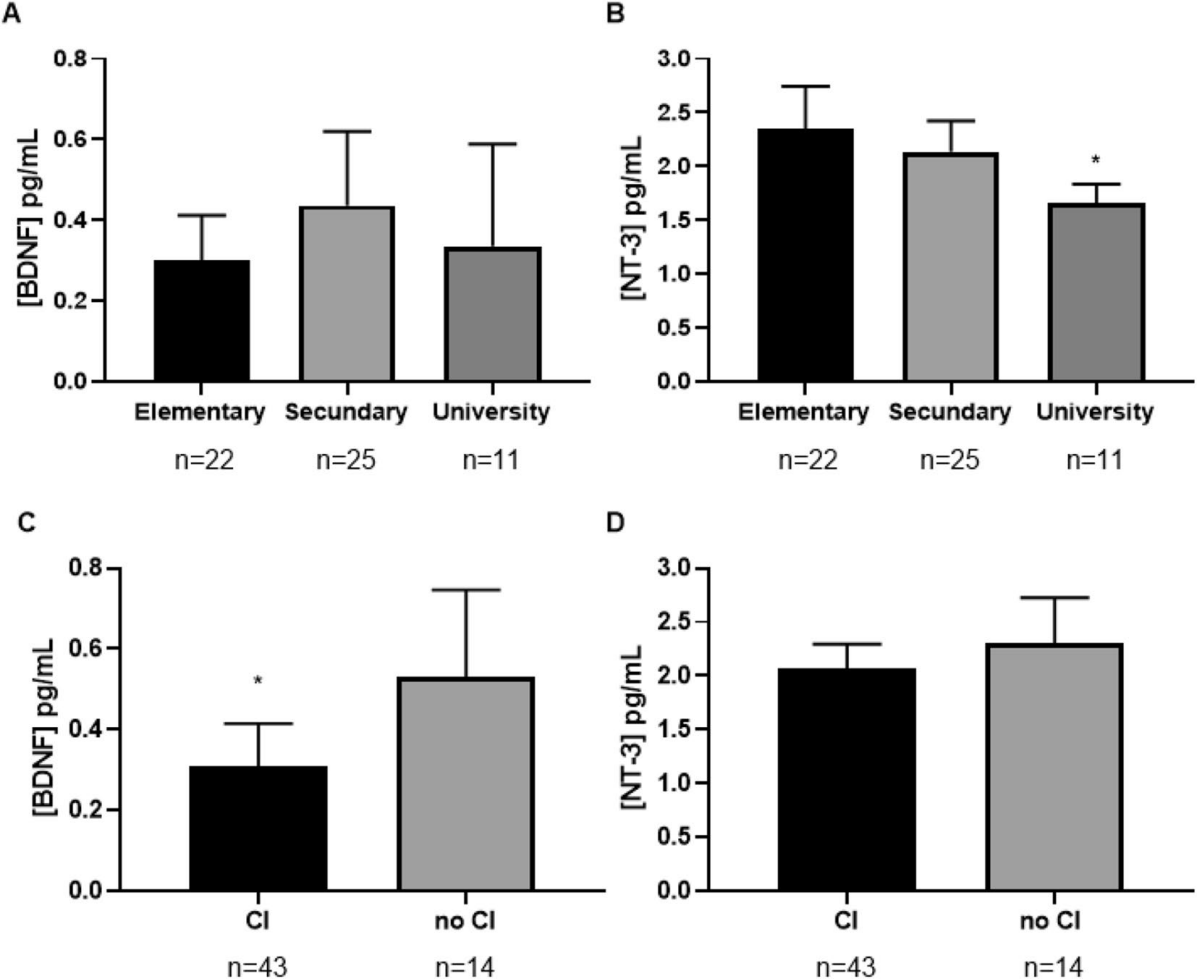
Plasma concentrations of either, BDNF or NT-3 in the sample according to education level and cognitive impairment evaluated using a two-way ANCOVA with "educational level" as a factor and "age" and "BMI” as covariates. Figure (**A**) shows that BDNF concentrations were not influenced by educational level. Figure (**B**) shows statistically significant differences in 3-NT concentrations between primary and university education levels. Figure (**C**) shows statistically significant differences in BDNF concentrations between patients with and without cognitive impairment. Figure (**D**) shows that NT-3 concentrations were not influenced by cognitive impairment. The bars are estimated marginal means and 95% confidence intervals. *p < 0.05. *CI* cognitive impairment.

As observed in Fig. 2A, plasma concentrations of 3-NT were significantly affected by the education level (F_2,58_ = 3.654, *p* = 0.033). Plasma concentrations of total 3-NT were significantly lower in patients with a university education level than in patients with a primary education level (*p* = 0.028). In contrast, as shown in Fig. 2B, education level did not affect plasma concentrations of BDNF (F_2,58_ = 0.147, *p* = 0.863), IGF-1 (F_2,58_ = 0.683, *p* = 0.509), IGF-2 (F_2,58_ = 2.662, *p* = 0.079) or IGFBP-3 (F_2,58_ = 1.144, *p* = 0.326).

### Cognitive impairment and plasma concentrations of BDNF, NT-3, IGF-1, IGF-2 and IGFBP-3 in alcohol-abstinent AUD patients

The effect of cognitive impairment on plasma levels of BDNF, 3-NT, IGF-1, IGF-2 and IGFBP-3 was evaluated using Student’s t test (see Supplementary Table S4). We confirmed that the data met the statistical assumptions, and logarithmic transformations were performed for values that did not meet the assumptions.

As Fig. 2C shows, plasma BDNF concentrations were significantly affected by the presence of cognitive impairment (t_56_ = 2.62, *p* = 0.011). Thus, plasma concentrations of total BDNF were significantly lower in patients with cognitive impairment than in patients without cognitive impairment. However, as Fig. 2D shows, cognitive impairment did not significantly affect plasma concentrations of 3-NT (t_56_ = 1.28, *p* = 0.206), IGF-1 (t_56_ = 0.294, *p* = 0.770), IGF-2 (t_56_ = 0.070, *p* = 0.944) or IGFBP-3 (t_53_ = 0.515, *p* = 0.609).

### Psychiatric comorbidity and plasma concentrations of BDNF, NT-3, IGF-1, IGF-2 and IGFBP-3 in alcohol-abstinent AUD patients

To determine whether the plasma concentrations of BDNF, NT-3, IGF-1, IGF-2 and IGFBP-3 in alcohol-abstinent AUD patients were influenced by the presence of psychiatric comorbidities, we used Student’s t test to examine the differences between patients with and without mood and anxiety disorders, as these were the most common psychiatric disorders among the study population (see Table 2). Plasma concentrations of total IGFBP-3 were significantly higher in patients with anxiety disorder than in patients without anxiety disorder (t_54_ = − 2.305, *p* = 0.025). However, we did not find statistically significant differences in the plasma concentrations of the other neurotrophic factors between patients with and without mood disorder (see Supplementary Tables S5 and S6).

### Predictive variables of cognitive impairment and education level in alcohol-abstinent AUD patients

First, to evaluate the predictive models using logistic regression, neurotrophic factors and relevant variables associated with alcohol consumption were included according to previous statistical analyses and were introduced using the *backward conditional* method.

**Figure 3:**
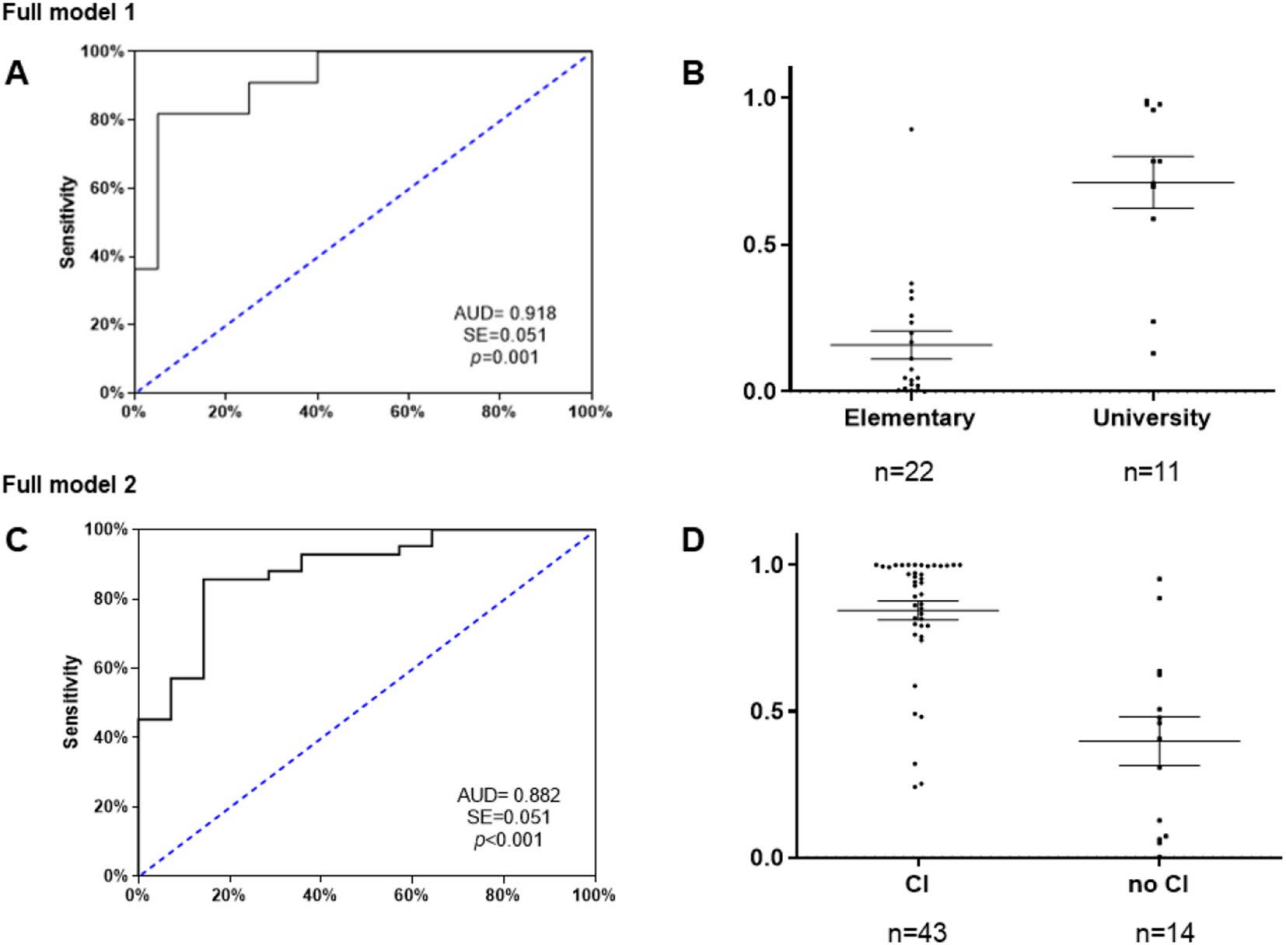
ROC analyses and scatter dots for multivariate predictive of full models of cognitive reserve (top, **A**, **B**) and cognitive impairment (down, **C**, **D**). ROC curves were generated by two binary regression logistic models using neurotrophic factors and alcohol-related variables as predictors following a backward stepwise entry method. (**A**) ROC curve for the full model 1 of cognitive reserve: “plasma concentrations of NT-3”, “age at onset of consumption” and “age at development of dependence”. (**B**) Scatter plot of the predictive probabilities for full model 1 of cognitive reserve (U = 18, *p* < 0.001). (**C**) ROC curve for the full model 2 of cognitive impairment: “plasma concentrations of BDNF”, “age at onset of consumption”, “length of AUD diagnosis”, “severity criteria”, and “duration of abstinence”. (**D**) Scatter plot of the predictive probabilities for the full model 2 of cognitive impairment (U = 69, p < 0.001). The lines of the scatterplots are means and standard deviations. *CI* cognitive impairment.

Full model 1 was performed to classify patients as having high or low cognitive reserve. The variables “plasma concentrations of NT-3”, “age at onset of consumption” and “age at development of dependence” were included in the model in the first step, and all three proved to be good predictor variables. Thus, we observed a new model with a good ability to discriminate between patients with a primary education and those who completed university studies (${X}^{2}$ = 4.800; *p* = 0.779) that was able to explain the variation in the dependent variable in 65.3% of cases, according to the Nagelkerke R2 method. Additionally, the new model had a classification rate of 90.3%, showing a very high sensitivity for the classification of patients with a primary education level (95%) and patients with a university education (81.8%). As shown in Fig. 3A, the ROC curve analysis showed an AUC = 0.918, which indicates high discriminatory power. As shown in Fig. 3B, the scatterplot of the predictive probabilities for patients with primary and university education indicated that the means were significantly different between the two groups (U = 18, *p* < 0.001).

Then, a binary logistic regression analysis was used to evaluate the potential for plasma 3-NT concentrations alone to be a good predictor for discriminating between patients with an elementary education and those with a university education. The final model showed a good ability to discriminate between patients with and without cognitive impairment (${X}^{2}$ = 1.609; p = 0.991) and was able to explain the variation in the dependent variable in 30.8% of cases, according to the Nagelkerke R2 method. On the other hand, we observed that the model had a classification percentage of 75.8%, showing high sensitivity for the classification of patients with a primary education (86.4%), but not for the classification of patients with a university education (54.5%). The ROC curve analysis showed an AUC = 0.793, which indicates average discriminatory power. The scatterplot of the predictive probabilities for patients with primary and university education indicated that the means were significantly different between the two groups (U = 50, *p* = 0.006).

Full model 2 was performed to classify patients with cognitive impairment from those without cognitive impairment. The variables “plasma concentrations of BDNF”, “age at onset of consumption”, “length of AUD diagnosis”, “severity criteria”, and “duration of abstinence” were included in the model in the first step and all were good predictors. Thus, we created a new model that exhibited a good ability to discriminate between patients with and without cognitive impairment (${X}^{2}$ = 6.801; p = 0.450) and could explain the variation in the dependent variable in 57.8% of cases, according to the Nagelkerke R2 method. Additionally, the new model had a classification percentage of 83.9%, showing average sensitivity for the classification of patients without cognitive impairment (66.7%) and high sensitivity for the classification of patients with cognitive impairment (90.2%). As shown in Fig. 3C, the ROC curve analysis showed an AUC = 0.882, which indicates high discriminatory power. As shown in Fig. 3D, the scatterplot of the predictive probabilities for patients with and without cognitive impairment indicated that the means were significantly different between the two groups (U = 69, p < 0.001).

Subsequently, a binary logistic regression analysis was used to evaluate the potential for plasma concentrations of BDNF to discriminate between patients with cognitive impairment from those without cognitive impairment. The final model showed a good ability to discriminate between patients with and without cognitive impairment (${X}^{2}$ = 11.370; *p* = 0.182) and was able to explain the variation in the dependent variable in 9.1% of cases, according to the Nagelkerke R2 method. Furthermore, the model had a classification percentage of 75.9%, showing high sensitivity for the classification of cognitively impaired patients (97.7%) but not for the classification of nonimpaired patients (13.3%). The ROC curve analysis showed an AUC = 0.731, which indicates average discriminatory power. The scatterplot of predictive probabilities for patients with and without cognitive impairment indicated that the means of the two groups were significantly different (U = 166, *p* = 0.009).

### Differential profiles associated with education level and growth factors in alcohol-abstinent AUD patients with and without cognitive impairment

**Figure 4:**
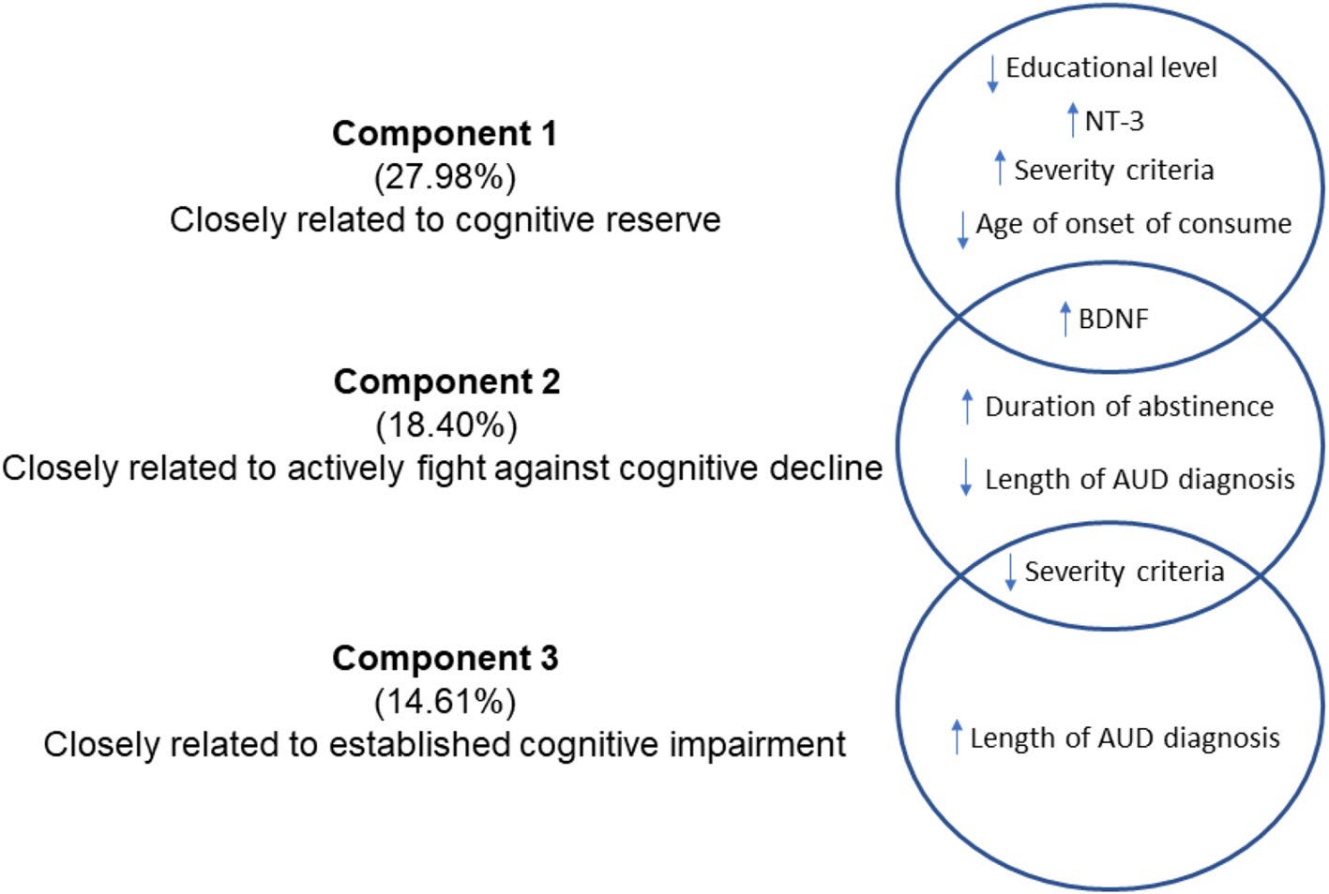
Exploratory principal component analysis in patients with cognitive impairment (n = 43). Three components (factors) together explained 60.98% of the variance associated with cognitive impairment in AUD patients.

To understand how all of these factors contribute to the differences between groups based on the presence of cognitive decline, a principal components analysis was performed. Three components together explained 60.98% of the variance associated with cognitive impairment in AUD patients (Fig. 4). Component 1 explained 27.98% of the total variance and was closely related to the cognitive reserve of the patients. Education level, NT-3, BDNF, severity of addiction and age at onset of consumption had a high factor load (− 0.691, 0.718, 0.346, 0.596, − 0.629, respectively). That is, this patient profile corresponds to those with a low educational level who started consuming alcohol early and who currently have high levels of 3-NT and BDNF and severe addiction. Component 2 explained 18.40% of the total variance and was closely associated with patients who are undergoing compensation or actively fighting against cognitive decline. BDNF, the severity of addiction, the duration of current abstinence and the duration since the AUD diagnosis had a high factor load (0.666, − 0.366, 0.711, − 0.388, respectively). Therefore, this patient profile corresponds to those who currently have higher levels of BDNF, lower severity of addiction, a longer duration of abstinence and a shorter duration of AUD. Lastly, component 3 explained 14.61% of the total variance and was related to patients with established cognitive impairment. The severity of addiction and the duration of consumption had a high factor load (− 0.438, 0.858, respectively). In other words, this profile corresponds to patients with a prolonged duration of AUD and a lower severity of addiction at the time of evaluation, possibly as a consequence of an established impairment.

## Discussion

Cognitive impairment and neurotrophic factors have been widely described in the scientific literature; however, they have never been linked in an integrated way with cognitive reserve and education level. The main results of this study indicate that (1) education level can act as a protective or risk factor in the onset of AUD and the development of cognitive impairment, (2) cognitive impairment is related to the onset of alcohol consumption and the length of AUD diagnosis throughout the lifespan and not to the patient’s psychiatric comorbidity, (3) the cognitive reserve is associated with frontal lobe functions but not with mnesic functions, (4) the plasma concentration of NT-3 is affected by cognitive reserve and can discriminate between patients with a high and low education level, (5) the plasma concentration of BDNF is affected by the state of frontal lobe function and is able to distinguish patients with and without cognitive impairment, (6) the cognitive reserve, NT-3 and BDNF are compensatory mechanisms for brain damage in the early stage of AUD, but not in later phases.

Education level is known to be a robust measure of cognitive reserve that contributes to delaying and smoothing the progress of mild cognitive impairment and other neurodegenerative pathologies^13,16^. In the field of addictions, cognitive reserve has been related to better cognitive performance and lower indicators of neurocognitive disorder^53,54^. The results of our study indicate that patients with a university education were protected against cognitive impairment caused by alcohol consumption throughout life, while patients with a primary education showed a special vulnerability to the occurrence of frontal deficits. Along this line, the consumption of high amounts of alcohol has been associated with worse cognitive performance in patients with low socioeconomic status^55^. Moreover, a longitudinal study indicated that having an education level lower than secondary school and a low-skill occupation are associated with an increased risk of dementia in people who consume alcohol^56^. It is important to mention that the differences in cognitive function found in our results were not due to psychiatric comorbidity, as was also described in another study^57^. In addition, in our study, cognitive reserve, understood as academic achievements, was related to frontal functions, but not with subjective memory failures. In agreement with this, a meta-analysis found that cognitive reserve is related to lateral and medial frontal areas, including anterior cingulate cortex and dorsolateral prefrontal cortex^18^. Thus, cognitive reserve has been associated with some domains of executive functions, such as working memory, verbal fluency and interference^58^.

On the other hand, the cognitive reserve of patients with substance use disorders has been related to the severity of the addictive process, the duration of abstinence and cognitive performance^53^. Similarly, our results indicate that patients with a low educational background initiated consumption and developed alcohol dependence early, while the opposite effect was observed for those who had a high education level. We think that the relationship between school dropout and substance use could be due to two factors. First, adolescents who consume alcohol are more likely to drop out of school. This is supported by the McAlaney study, in which the negative consequences most frequently reported by European substance-consuming students were skipping classes, memory problems and poor academic performance^59^. Second, dropping out of school could predispose students to begin consuming alcohol since they are deprived of the protection offered by the educational system and additional years of schooling. Along this line, according to Crum, young people who drop out of high school or college have a higher risk of developing alcohol abuse in adulthood than those who complete college or high school^60^. Regardless of the cause or consequence, repeated alcohol poisoning in adolescence and a lack of cognitive stimulation interfere with brain development, increasing subsequent neuropsychiatric vulnerability^61,62^.

Regarding alcohol-related cognitive impairment, the early onset of alcohol consumption seems to be a risk factor for poor subsequent neuropsychological functioning in young adults^63^. Likewise, our results indicate that patients with cognitive impairment were more likely to have started consuming alcohol early and to have had a longer duration of AUD. Thus, they are patients who are at a more advanced stage of the disorder and have the added possibility of experiencing a liver complication in the future. It is interesting to note that patients with alcohol-related cirrhotic disease show greater cognitive deficits and worse brain reserve than patients with non-alcoholic cirrhosis^64^. In addition, patients with cirrhosis who have a high cognitive reserve have a better quality of life, while patients with cirrhosis who have covert encephalopathy show a lower cognitive reserve^65^. On the other hand, the patients in our study without cognitive impairment had a higher number of addiction severity criteria and a shorter duration of abstinence at the time of evaluation, indicating that they were patients who were actively fighting the disorder. This finding demonstrates that cognitive impairment is not associated with the amount of alcohol consumption but rather with the onset of consumption and the duration of AUD throughout life^63^. In addition, this result indicates that cognitive function does not improve with the duration of abstinence and may indicate permanent damage to cognitive abilities in alcohol-consuming patients, as indicated by other studies^35,66,67^.

On the other hand, clinical and preclinical studies have widely described that alcohol produces a proinflammatory condition in the central nervous system^26,68^. Microglia respond to alcohol through TLR4, which activates signaling cascades, including the NF-KB and MAPK pathways, which induce the activation of proinflammatory mediators^29,69^, to the detriment of trophic signaling and cognition^28,70^. Previously, our team found decreases in plasma concentrations of NT-3 and BDNF and an association between frontal functions and circulating BDNF in patients with AUD^46,50^. In light of our results, we can say that the decrease in the BDNF and 3-NT levels of AUD patients does not interfere with frontal function in those with higher education, probably because cognitive reserve compensates for these biochemical deficits. However, the decrease in the levels of BDNF and 3-NT seems to trigger cognitive decline in patients with low education levels because their low cognitive reserve prevents them from compensating for organ damage derived from alcohol consumption. The fact that 3-NT levels are higher in patients with a low education level than in those with a high education level could reveal the failed attempt of the system to compensate for the brain damage with neurogenesis instead of implementing more effective compensatory mechanisms that depend on a higher cognitive reserve.

In terms of growth factors, BDNF seems to be a candidate for describing the neurobiological basis of cognitive reserve^71^. Physical activity, social interactions, cognitive stimulation, a high education level and an enriching environment have been associated with increases in BDNF levels and a lower risk of dementia^44,72–75^. In contrast, Val66 Met polymorphism is known to prevent the release of mature BDNF, disrupt cognitive functioning^76–78^ and interfere with the protective effect of cognitive reserve on executive functions^79^. However, it seems that the scientific literature has underestimated the role of 3-NT in cognitive processes in adult life.

In our study, 3-NT and BDNF were shown to be robust factors of brain damage and cognitive reserve, respectively, that allowed us to discriminate very clearly between cognitively impaired and non-impaired patients and between patients with high and low cognitive reserve. In addition, we have shown the compensatory role that these two neurotrophins have on cognitive decline in the early phase of AUD. Thus, in a subgroup of patients, we observed that NT-3 and BDNF signaling is initiated to compensate for the damage caused by alcohol in patients with a low cognitive reserve who started drinking early. Second, in another subset of patients, we showed that the compensatory signal of BDNF is also activated independently of the cognitive reserve in the early stage of AUD. In contrast, in a third subset of patients, we observed that neurotrophic signaling and the protective effect of cognitive reserve disappear in the advanced phase of AUD. Therefore, these findings likely indicate that BDNF levels decrease when organ damage is already established. In line with our results, there is an emerging line of research that supports the role of the cognitive reserve and the BDNF/TrkB signaling pathway as compensatory responses that delay symptomatology in the early stage of Alzheimer's disease but cannot prevent neurodegeneration in more advanced phases^16,80–82^. Although BDNF has not been shown to be able to differentiate among different neurodegenerative diseases^83^, it does seem to be a good predictor of the severity and progression of cognitive impairment^74,84^.

In conclusion, educational history and a simple cognitive evaluation, together with the measurement of BDNF, allowed us to identify patients who are at risk of cognitive impairment and differentiate them from those who are already cognitively impaired, stratifying them in a very clear way. Such information allows interventions to be personalized according to the stage of the neuropathology, such as by recommending cognitive and physical stimulation for less affected people or establishing palliative treatment in more severe cases. Additionally, this knowledge should focus attention on early care measures in anticipation of future needs since school dropout and alcohol consumption lead to the impairment of cognitive abilities in adult life.

### Limitations and future prospects

This study has a number of limitations that future research should take into account. The study has a small sample of patients and lacks significant representation of the female population, which prevents the investigation of gender/sex differences in educational level, cognitive impairment and neurotrophic factors. Finally, the study did not include another relevant growth factor, Nerve Growth Factor (NGF) that might be accounting for the cognitive impairment described in AUD patients, and that should be analyzed in future studies.

## Methods

### Recruitment and screening of participants

The study was conducted based on a cohort of Addictive Disorders Network (RTA, for its initials in Spanish) patients with AUD recruited from 2016 to date. We relied on a database with a total of 262 alcohol-abstinent patients in outpatient treatment, 148 of whom had undergone measurements of neurotrophic factors in blood plasma. Of these 148 patients, we included 58 patients who had undergone a brief neuropsychological evaluation using the Frontal Assessment Battery (FAB) and Memory Failures of Everyday questionnaire (MFE), which will be described below. The patients were recruited from the Psychiatry Service of the *12 de Octubre University Hospital* (Madrid, Spain) and the *Provincial Drug Addiction Center* (Málaga, Spain).

To be eligible for the study, subjects has to meet eligibility criteria based on (A) inclusion criteria: age ≥ 18 years up to 65 years of age, lifetime AUD, and at least 2 weeks of abstinence before testing confirmed by repeated negative breathalyzer test; (B) exclusion criteria: personal history of chronic diseases (e.g. cardiovascular, respiratory, renal, hepatic, neurological or endocrinological diseases), personal history of autoimmune disorders, cancer, presence of chronic viral infectious diseases (VIH, HB, HC), incapacitating cognitive alterations to complete psychiatric interview and neuropsychological evaluation, and pregnancy for female participants.

### Ethical declaration

Written informed consent was obtained from each participant after a complete description of the study was provided. All participants had the opportunity to discuss any questions or problems. The study and the protocols for recruitment were approved by the Ethics Committee of the Regional University Hospital of Málaga in accordance with the Ethical Principles for Medical Research involving Human Subjects adopted in the Declaration of Helsinki by the World Medical Association (64th General Assembly of the WMA, Fortaleza, Brazil, October 2013) and Recommendation no. R (97) 5 of the Committee of Ministers to the Member States on the protection of medical data (1997), the Spanish law on data protection [Regulation (EU) 2016/679 of the European Parliament and of the Council of April 27, 2016 on the protection of natural persons with respect to the processing of personal data and the free circulation of such data, and which repeals Directive 95/46/EC (General Data Protection Regulation)]. All collected data received code numbers to maintain privacy and confidentiality.

### Psychiatric and neuropsychological evaluation

The Spanish version of the PRISM (Psychiatric Research Interview for Substance and Mental Diseases) diagnostic interview was used for the evaluation of substance use disorders and other psychiatric disorders in accordance with to the criteria of the DSM-IV-TR (Diagnostic and Statistical Manual of Disorders Mental, 4th edition, text review). The PRISM is a semistructured interview with good psychometric properties for the evaluation of substance use disorders and the main psychiatric disorders comorbid with substance use^85,86^.

The neuropsychological evaluation was performed using two different tests that have proven reliability and good psychometric properties: the Spanish version of the FAB, which was used to diagnose frontal dysfunction^51^ and the MFE, which was used to evaluate daily memory failures^52^. The total FAB score ranges from 0 to 18, and the test evaluates the subdomains prehension behavior, go-no go, conflicting instructions, lexical fluency and Luria’s motor series. A cutoff score lower than 16 differentiates normal frontal function from mild deficits, and a score below the cutoff score of 13 differentiates mild and severe frontal lobe dysfunction^51^. The MFE questionnaire consists of 30 items and is useful for evaluating memory failures in daily life. Cognitive complaint scores of less than eight points correspond to optimal memory functioning, scores between 8 and 35 points are equivalent to normal functioning with insignificant memory failures, scores between 36 and 50 indicate deterioration in memory function with some impact on everyday life, and scores above 50 points correspond to moderate or severe deterioration with substantial impact on daily functioning^52^.

### Obtaining plasma samples

Blood samples were obtained in the morning after an 8- to 12-h fast (before psychiatric interviews). Venous blood was extracted into 10-ml K2 EDTA tubes (BD, Franklin Lakes, NJ, USA) and immediately processed to obtain plasma. Blood samples were centrifuged at 2200×*g* for 15 min (4 °C) and analyzed individually for infectious diseases using three rapid commercial tests for HIV, hepatitis B and hepatitis C (Strasburg, Cedex, France). Finally, the plasma samples were aliquoted, recorded and stored individually at − 80 °C until further analysis.

### Analysis of neurotrophic factors

Plasma levels of BDNF, IGF-2 and NT-3 were determined using different enzyme-linked immunosorbent assays (ELISA) according to the manufacturer's instructions: the human BDNF SimpleStep^®^ ELISA kit (# ab212166, Abcam, Cambridge, UK), the Quantikine Human IGF-2 ELISA Kit (# DG200, R&D Systems, Minneapolis, MN, USA) and the NT-3 ELISA Kit (# EHNTF3, Thermo Fisher Scientific, Alcobendas, Madrid, Spain). To perform the ELISA protocols, we used 50 μL of plasma, as previously described^50^. Plasma concentrations of IGF-1 and IGFBP-3 were estimated by double antibody radioimmunoassay, as described in^39^. Plasma fractions were incubated with 125I-IGF-1 at 4 °C for 24 h with IGF-1 antiserum (UB2-495). The plasma concentration of IGFBP-3 was determined in duplicate by RIA using a commercially available kit (Mediagnost GmbH, Reutlinger, Germany) according to the manufacturer's instructions. IGFBP-3 concentrations were expressed as μg/mL. A calibration curve and internal controls were included in each test.

### Statistical analysis

All data in the tables are expressed as numbers, percentage of subjects [N (%)], means and standard deviations (SD). The significance of the differences for qualitative variables and normal continuous variables was determined using Fisher’s exact test (chi-square) and Student’s t test, respectively.

Statistical analyses of the FAB and MMSE cognitive scores and concentrations of BDNF, 3-NT, IGF-1, IGF-2 and IGFBP-3 were performed using a univariate analysis of covariance (ANCOVA) to determine the relative effects of education level when covariates such as age and body mass index (BMI) were controlled. Post hoc tests for multiple comparisons were performed using the Bonferroni correction.

Before the continuous variables were included in these analyses, the assumptions of normality and homoscedasticity were confirmed. The normal distribution of the variables was evaluated using the Shapiro–Wilk test. Logarithmic (10) transformations of the variables with nonnormal distribution were used to preserve the parametric assumptions of distributions with positive bias and estimated marginal means [95% confidence interval (95% CI)]. The equality of variances was tested with the Levene test, using Welch’s T in cases of noncompliance.

To identify the variables that were able to discriminate between impaired and nonimpaired patients and between patients with an elementary and a university education, a binary logistic regression analysis was performed using Pearson’s chi-square test (χ^2^), and the results satisfied the Hosmer–Lemeshow test. Sociodemographic variables, variables related to cocaine use patterns and psychiatric comorbidity variables were included in the equation.

Lastly, an exploratory factor analysis with varimax rotation and bivariate relationships (correlation) was performed to determine the different profiles of alcohol-abstinent patients with cognitive decline. Only variables with a factor load of at least 0.3 (i.e., those that share at least 10% of the variance with a factor) were used for the interpretation. A *p* value less than 0.05 was considered statistically significant.

Statistical analyses were performed using GraphPad Prism version 5.04 and IBM SPSS Statistical version 22 (IBM, Armonk, NY, USA). A value of p < 0.05 was considered statistically significant.

## Supplementary Information

Supplementary Information.

## Data Availability

The data that support the findings of this study are available on reasonable request from the corresponding author.
