# Supplementary material for: Evaluation of neurotrophic factors and education level as predictors of cognitive decline in alcohol use disorder
Source: Sci Rep. 2021 Aug 2;11:15583. doi: 10.1038/s41598-021-95131-2 (PMC8328971; doi:10.1038/s41598-021-95131-2)
Supplement: Supplementary file 1 — Supplementary Information. [file 41598_2021_95131_MOESM1_ESM.pdf]

SUPPLEMENTARY MATERIALS

Additional statistical analysis showing:

1. **The lack of relationship in between cognitive impairment and a) mood disorders (table S1), and b) anxiety disorders (table S2).**

2. **The relationship of circulating neurotrophic factors (BDNF, NT-3, IGF1, IGF-2 and IGFBP-3) with a) Education Level (table S3), b) the presence of cognitive impairment (table S4), c) the presence of mood disorder (table S5), d) the presence of anxiety disorder (table S6).**

Supplementary Table S1. Cognitive impairment according to the presence of Mood Disorder.

SUBCOHORT (N=56)					
VARIABLE	MOOD DISORDER N=25	NO MOOD DISORDER N=31	t-test (Statistics)		
			t-value	df	*p* value
**FAB Score** *[Mean (SD)]*	14.80 (2.43)	15.16 (1.83)	0.635	54	0.528
**MFE-30** *[Mean (SD)]*	32.48 (16.21)	28.45 (17.64)	-0,881	54	0.328

Abbreviations: FAB, frontal assesmet battery score, MFE-3, memory failures of everyday questionnaire; SD, standard deviation, df=degree of freedom

Supplementary Table S2.Cognitive impairment according to the presence of Anxiety Disorder.

SUBCOHORT (N=56)					
VARIABLE	ANXIETY DISORDER N=20	NO ANXIETY DISORDER N=36	t-test (Statistics)		
			t-value	df	*p*-value
**FAB Score** *[Mean (SD)]*	15.15 (2.16)	14.92 (2.10)	-0.394	54	0.695
**MFE-30** *[Mean (SD)]*	33.10 (19.23)	28.67 (15.65)	-0.935	54	0.354

Abbreviations: FAB, frontal assesmet battery score, MFE-3, memory failures of everyday questionnaire; SD, standard deviation, df=degree of freedom

Supplementary Table S3.Plasma concentration of neurotrophic factors according to the education level.

SUBCOHORT (N=58)						
VARIABLES	ELEMENTARY N=22	SECONDARY N=25	UNIVERSITY N=11	ANCOVA (Statistics)[1]		
				F-value	df	*p*-Value
**BDNF (pg/mL)** *[Mean (95%CI)]*	0.322 [0.176]	0.416 [0.279-0.554]	0.333 [0.126-0.539]	0.147	2,58	0.863
**NT-3 (pg/ml)** *[Mean (95%CI)]*	2.365 [2.058-2.671]	2.116 [1.828-2.403]	1.679 [1.247-2.111]	3.654	2,58	**0.033**
**IGF-1 (ng/mL)** *[Mean (95%CI)]*	141.095 [115.280-166.909]	163.794 [139.587-188.001]	160.085 [123.692-196.478]	0.683	2,58	0.509
**IGF-2 (ng/mL)** *[Mean (95%CI)]*	327.562 [290.116-365.008]	374.970 [339.856-410.084]	312.819 [260.028-365.611]	2.662	2,58	0.079
**IGFBP-3 (μg/mL)** *[Mean (95%CI)]*	2.984 [2.560-3.408]	3.422 [3.024-3.819]	3.165 [2.567-3.763]	1.144	2,58	0.326

[1]Statistical analysis was conducted on the logarithmic transformed values to ensure statistical assumptions for BDNF, NT-3 and IGF-2 concentrations. Abbreviations: df=degree of freedom

Supplementary Table S4. Plasma concentration of neurotrophic factors according to the presence of cognitive impairment.

SUBCOHORT (N=57)					
VARIABLES	COGNITIVE IMPAIRMENT N=43	NO COGNITIVE IMPAIRMENT N=14	t-test (Statistics)[1)]		
			t-value	df	*p*-Value
**BDNF (pg/mL)** *[Mean (SD)]*	0.2066 (0.750)	0.2303 (0.762)	0.2630	56	**0.011**
**NT-3 (pg/mL)** *[Mean (SD)]*	0.308 (0.348)	0.529 (0.391)	1.319	56	0.192
**IGF-1 (ng/mL)** *[Mean (SD)]*	154.797 (60.30)	153.573 (37.670)	-0.085[2)]	45.431	0.933
**IGF-2 (ng/mL)** *[Mean (SD)]*	343.207 (102.381)	350.916 (63.226)	0.273	56	0.786
**IGFBP-3 (μg/mL)** *[Mean (SD)]*	3.144 (1.176)	3.387 (0.588)	1.032[2)]	48.725	0.307

[1)]Statistical analysis was conducted on the logarithmic transformed values to ensure statistical assumptions for BDNF and NT-3 concentrations. [2)]Welch's t-test. Abbreviations: df=degree of freedom

Supplementary Table S5. Plasma concentration of neurotrophic factors according to the presence of Mood Disorder.

SUBCOHORT (N=56)					
VARIABLES	MOOD DISORDER N=25	NO MOOD DISORDER N=31	t-test (Statistics)[1]		
			t-value	df	*p*-Value
**BDNF (pg/mL)** *[Mean (SD)]*	0.323 (0.277)	0.418 (0.433)	0.258	54	0.798
**NT-3 (pg/mL)** *[Mean (SD)]*	2.004 (0.785)	2.242 (0.741)	1.439	54	0.156
**IGF-1 (ng/mL)** *[Mean (SD)]*	165.672 (72.327)	149.802 (51.545)	-0.958	54	0.343
**IGF-2 (ng/mL)** *[Mean (SD)]*	353.913 (103.620)	324.643 (84.671)	-0,397	54	0.693
**IGFBP-3 (μg/mL)** *[Mean (SD)]*	3.464 (1.1195)	3.065 (0.917)	-1.247	54	0.218

[1)]Statistical analysis was conducted on the logarithmic transformed values to ensure statistical assumptions for BDNF, NT-3 and IGF-2 and IGFBP-3 concentrations. Abbreviations: df=degree of freedom

Supplementary Table S6. Plasma concentration of neurotrophic factors according to the presence of Anxiety Disorder.

SUBCOHORT (N=56)					
VARIABLES	ANXIETY DISORDER N=20	NO ANXIETY DISORDER N=36	t-test (Statistics)[1]		
			t-value	df	*p*-Value
**BDNF (pg/mL)** *[Mean (SD)]*	0.361 (0.269)	0.383 (0.421)	-0.457	54	0.649
**NT-3 (pg/mL)** *[Mean (SD)]*	2.175 (0.959)	2.113 (0.644)	-0.072	54	0.943
**IGF-1 (ng/mL)** *[Mean (SD)]*	166.952 (71.559)	151.295 (55.625)	-0.919	54	0.367
**IGF-2 (ng/mL)** *[Mean (SD)]*	351.380 (67.758)	345.616 (105.135)	-0,221	54	0.826
**IGFBP-3 (μg/mL)** *[Mean (SD)]*	3.650 (0.874)	3.017 (1.041)	-2.305	54	**0.025**

[1]Statistical analysis was conducted on the logarithmic transformed values to ensure statistical assumptions for BDNF and NT-3 concentrations. Abbreviations: df=degree of freedom
